# Supplementary material for: Efficacy of ceiling-mounted mosquito nets for malaria vector control in a Peruvian Amazon riverine community: A stepped-wedge cluster randomized trial
Source: PLoS One. 2025 Oct 21;20(10):e0325089. doi: 10.1371/journal.pone.0325089 (PMC12539722; doi:10.1371/journal.pone.0325089)
Supplement: S1 File — This file includes the official ethics approval certificate (Certificate No. 08-CIEI-HRL-2024) issued by the Loreto Regional Hospital Ethics Committee and the complete protocol for the stepped-wedge cluster randomized trial evaluating ceiling-mounted mosquito nets in the Llanchama community. The protocol details study objectives, design, sampling strategy, data collection and analysis methods, ethical considerations, and includes informed consent templates for participants and field collectors. (PDF) [file pone.0325089.s001.pdf]

S1 File. Ethics approval certificate and detailed study protocol

"HOSPITAL REGIONAL DE LORETO "FELIPE ARRIOLA IGLESIAS"

CONSTANCIA No 08– CIEI – HRL – 2024

El director del Hospital Regional de Loreto; a través de la Oficina de Apoyo a la Docencia e Investigación, y el Comité Institucional de Ética e Investigación (CIEI), **HACE CONSTAR** que el presente proyecto de Investigación, consignado líneas abajo, fue **APROBADO**, en cumplimiento de los estándares del Instituto Nacional de Salud (INS), acorde con las prioridades Regionales de Investigación, Balance Riesgo/beneficio y confiabilidad de los datos, entre otras. Siendo catalogado como: **ESTUDIO CLÍNICO SIN RIESGO**, según detalle:

Título del Proyecto:	**ESTUDIO EXPERIMENTAL DE DENSIDAD DE VECTORES EN VIVIENDAS RENOVADAS CON MALLA MOSQUITERO, COMUNIDAD DE LLANCHAMA, DISTRITO DE SAN JUAN BAUTISTA, PROVINCIA DE MAYNAS, REGIÓN LORETO.**
Código de Inscripción:	**ID-008-CIEI-2024.**
Modalidad de investigación:	**EXTRA INSTITUCIONAL.**
Investigador (es):	**CINTHIA INTI QUIROZ.**

Cualquier eventualidad durante su ejecución, los investigadores reportarán de acuerdo con las Normas y plazos establecidos, así mismo emitirán el informe final socializando los RESULTADOS obtenidos. El presente documento tiene vigencia hasta el 19 de febrero del 2025. **El trámite para su renovación será un mínimo de 30 días antes de su vencimiento**.

Punchana, 19 de febrero del 2024.

Gerencia Regional de Salud-Loreto
Hospital Regional de Loreto
"Felipe Arriola Iglesias"
DR. CESAR JOHNNY [illegible] ASAYAG
CMP. Nº 26 [illegible] RNE. 22676
Director General

CJRA/GNRM/JLGP.

Escaneado con CamScanner

(TRANSLATION FROM SPANISH TO ENGLISH)

LORETO REGIONAL HOSPITAL "FELIPE ARRIOLA IGLESIAS"

CERTIFICATE No. 08-CIEI-HRL-2024

The director of the Loreto Regional Hospital, through the Teaching and Research Support Office and the Institutional Ethics and Research Committee (CIEI), CERTIFIES that the present research project, listed below, was APPROVED, in compliance with the standards of the National Institute of Health (INS), in accordance with the Regional Research Priorities, Risk/Benefit Balance, and data reliability, among others. Classified as a RISK-FREE CLINICAL STUDY, as detailed below:

Project Title:	EXPERIMENTAL STUDY OF VECTOR DENSITY IN HOUSING RENOVATED WITH MOSQUITO NETTING, LLANCHAMA COMMUNITY, SAN JUAN BAUTISTA DISTRICT, MAYNAS PROVINCE, LORETO REGION
Registration Code:	ID-008-CIEI-2024
Research Modality:	EXTRA-INSTITUTIONAL
Researcher(s):	CINTHIA INTI QUIROZ

In case of any eventuality during the execution, the researchers will report in accordance with the established standards and deadlines, and will also issue the final report socializing the results obtained. This document is valid until February 19, 2025. The renewal process will be completed at least 30 days before its expiration.

Punchana, February 19, 2024

Loreto Regional Health Department
Felipe Arriola Iglesias Regional Hospital of Loreto

Dr. César Johnny Ramal Asayag
CMP No. 26491, RNE No. 22676
General Manager

ESTUDIO EXPERIMENTAL DE DENSIDAD DE VECTORES EN VIVIENDAS RENOVADAS CON MALLA MOSQUITERO, COMUNIDAD LLANCHAMA, DISTRITO DE SAN JUAN BAUTISTA, PROVINCIA DE MAYNAS, REGIÓN LORETO

Elaborado por:

World Vision Perú

Gerencia Regional de Salud de Loreto

KOICA

SETIEMBRE 2023

Contenido

1 Antecedentes 3
2 Propuesta del proyecto 3
3 Objetivo del estudio 4
3.1 Objetivo general 4
3.2 Objetivos específicos 4
4 Metodología 4
4.1 Diseño del estudio 4
4.2 Población y muestra 5
4.3 Técnica de muestreo 5
4.4 Criterios de exclusión 6
4.5 Procedimiento de recolección de información 6
4.6 Procesamiento y análisis de información .... **¡Error! Marcador no definido.**
4.7 Socialización de resultados 7
4.8 Propiedad intelectual 7
5 Consideraciones éticas 7
6 Presupuesto 8
7 Cronograma 8
8 Anexos 9

1 Antecedentes

A pesar de la reducción de la mortalidad por Malaria en el mundo en un 60% durante el período 2000 a 2019, ésta es aún muy alta e inaceptable. Hasta abril del 2023 en la Amazonía peruana, en el departamento de Loreto, el número de casos por malaria fue de 11,163.

Desde el 2018, se implementa el Plan de Eliminación de Malaria, denominado Plan Malaria Cero (PMC) con una vigencia hasta el 2021 y hasta el año 2020, había logrado disminuir los casos en un 75% en la región Loreto y ha evitado más de 80 mil casos en este periodo.

Sin embargo, debido a la atención de la pandemia por COVID-19 algunas actividades fueron descontinuadas o disminuyeron. Por lo tanto actualmente, las acciones para la eliminación de la malaria se encuentran integradas en el Plan Estratégico Multisectorial al 2030 de la Política Nacional Multisectorial de Salud "Perú, País Saludable", teniendo como objetivo al 2030, disminuir en 90% la malaria en el Perú.

Entre las alternativas de solución planteadas por el gobierno y que vienen ejecutándose en el departamento de Loreto se incluyen: identificación de casos a través de la detección pasiva y búsqueda activa de casos, detección y diagnóstico de casos dentro de las 24 horas de iniciado el cuadro clínico, formación de agentes comunitarios y trabajadores de salud, vigilancia epidemiológica, disminuir el riesgo del contacto entre el hombre y el vector a través del uso de mosquiteros tratados con insecticidas (MTI), aplicación de rociado residual intradomiciliario, ordenamiento del medio, articulación intergubernamental, intersectorial y la participación comunitaria, desarrollo de un plan comunicacional y monitoreo incorporando un equipo Ad hoc a nivel regional.

Todas estas acciones son parte de los cuatros componentes (diagnóstico temprano, tratamiento oportuno, investigación y respuesta) de la estrategia del Plan hacia la Eliminación de la malaria al 2030.

2 Propuesta del proyecto

Este estudio se ejecuta en el marco del Proyecto "Reducción de la Carga de Enfermedades Tropicales Desatendidas y promoción de la Salud Básica en la Amazonía Peruana" implementado por la ONG World Vision Perú, en asocio con KOICA, para el periodo 2022-2024, en la jurisdicción del distrito de San Juan Bautista, provincia de Maynas, región Loreto. Por lo que, los hallazgos de este estudio nos permitirá confirmar si la estrategia de renovación de viviendas con malla mosquitero, nos permite disminuir el riesgo de contacto entre el hombre y vector y así evitar los casos de malaria.

El proyecto como meta espera contribuir a la reducción de la tasa de incidencia de dengue y malaria en el distrito de San Juan Bautista, para ello se tiene las siguientes acciones claves:

- Fortalecer el sistema de salud: a través del desarrollo de capacidades en el personal de salud, implementación de insumos y equipos para el diagnóstico temprano de malaria y dengue, mejorar la calidad de la información epidemiológica accediendo al servicio de internet por medio de instalación de radio enlaces y ampliar el servicio de salud hacia la comunidad para la búsqueda activa de casos a través de campañas de salud itinerantes.

- Influencias en el cambio de comportamiento de la comunidad, para que adopten medidas preventivas que eviten la enfermedad por dengue y malaria a través del desarrollo de capacidades a agentes comunitarios de salud, difusión de mensajes preventivos usando medios masivos, contribuir en la cobertura de viviendas fumigadas según comunidades priorizadas, promover medidas de prevención en las escuelas y probar la estrategia de renovación de viviendas usando malla mosquitero como medida de prevención de malaria.

Esta última, es la razón de este estudio y se espera utilizar sus hallazgos para la incidencia local, regional, y/o nacional y promoción de salud en la comunidad.

3 Objetivo del estudio

3.1 Objetivo general

- Determinar si la renovación de viviendas con malla mosquitero tiene efecto en la disminución del vector Anopheles dentro de la vivienda.

3.2 Objetivos específicos

- Determinar el efecto de renovar las viviendas con la colocación de mallas mosquitero en el cielo raso de la vivienda en la disminución del vector Anopheles dentro de la vivienda.
- Determinar el efecto de renovar las viviendas con la colocación de mallas mosquitero en el cielo raso de la vivienda en la disminución del vector Anopheles fuera de la vivienda.
- Determinar las pautas para la renovación de viviendas según sus tipos, colocando malla mosquitero como factor para la reducción de la densidad del vector Anopheles y otros hematófagos.
- Determinar el nivel de riesgo de transmisión de malaria según indicadores del vector Anopheles y otros hematófagos.
- Determinar la tasa de incidencia acumulada de malaria a través de la medición del Índice Parasitario Anual (IPA).
- Determinar la temperatura y humedad relativa dentro, fuera y lejos de la vivienda.

- Determinar los horarios y número de horas de permanencia de los miembros de la familia dentro y fuera de la vivienda.
- Determinar horarios y número de horas de permanencia de los miembros de la familia dentro del mosquitero tratados con insecticida de larga duración.

4 Metodología

4.1 Diseño del estudio

Este estudio seguirá un diseño de tipo experimental aleatorizado de grupos escalonados (stepped wedge cluster randomised trial) con el objetivo de evaluar el evaluar el efecto de la colocación de mallas mosquitero en el cielo raso de las viviendas en la disminución de la densidad del vector Anopheles , en la comunidad de Llanchama, distrito de San Juan Bautista, Loreto, Perú.

La malaria en este distrito tiene un patrón epidemiológico inestable y un comportamiento estacional, con un pico entre los meses de marzo a agosto. Todos los grupos etarios son afectados y la mayor parte de los casos son sintomáticos. El principal vector de la malaria es el *Anopheles darlingi*, pero también se han reportado la circulación de *Anopheles*

benarrochi en la región.[1] Por esta razón se previsto que el estudio se ejecute en el periodo de un año e incluirá una evaluación basal y cuatro escalamientos trimestrales.

4.2 Población y muestra

La población de estudio estará compuesta por toda la comunidad de Llanchama, la misma que cuenta con un total de 85 viviendas con residentes permanentes. Estas serán intervenidas de manera escalonada a razón de 19 casas, las cuales serán tomadas al azar hasta completar la intervención en no menos del 67% de las casas de la comunidad (Gráfico 1).

Figura 1. Secuencia escalonada del estudio

Secuencia	Basal	Control 1	Control 2	Control 3
Secuencia 1	19	19	19	19
Secuencia 2	19	19	19	19
Secuencia 3	19	19	19	19
Total de Viviendas	57 (57 vs 0)	57 (38 vs 19)	57 (19 vs 38)	57 (0 vs 57)

* Blanco, Control; Gris, Intervención

Como el objeto de controlar por tipo de casa el muestreo será ponderado a fin de enrolar una misma proporción de casas de los tipos A, B, C y D en cada secuencia del estudio y procurar representar la distribución de casas según tipo de vivienda en la comunidad, las cuales se distribuyen a razón de 10%, 20%, 30% y 40%, aproximadamente.

Asumiendo un poder de estudio del 80%, un alfa de 0,05, un efecto esperado de una reducción del 50% del conteo de *Anopheles darlingi* picando en el interior de las casas, se estimó que se requerían un mínimo de 57 casas en un diseño de tipo experimental aleatorizado de grupos escalonados (a razón de 19 casas por escalón) con un basal y tres controles.

4.3 Técnica de muestreo y enrolamiento

Tomando en consideración las características de las viviendas de los casos y controles y sus tipos, según la categorización hecha por la Dirección de Salud Ambiental, se las identificará de acuerdo con la lista de viviendas de la comunidad de Llanchama, las cuales serán georreferenciadas previamente y se enumerará según sus ubicaciones en el mapa comunitario.

A cada jefe de familia de las viviendas seleccionadas, se les informará sobre el estudio y en caso de no aceptar participar por cualquier razón, esta será reemplazada de forma aleatoria por otra vivienda que tenga las mismas características.

Cada familia firmará de antemano un consentimiento informado y accederá a un documento y explicación detallada sobre la colecta de vectores mediante la técnica de cebo humano y otros alcances del estudio. Así mismo durante el proceso se podrá atender

cualquier duda y será libre de cambiar de opinión y retirarse del estudio en cualquier momento.

4.4 Criterios de exclusión

Se excluirán del estudio todas aquellas viviendas que hayan sido nebulizadas o recibido rociamiento residual intradomiciliario en los últimos 90 días.

También aquellas viviendas del grupo de casos que no hayan hecho la renovación de su vivienda de forma completa serán excluidas del estudio.

4.5 Procedimiento de recolección de información

Basado en la experiencia del piloto de renovación de viviendas en el 2022, se ha establecido el primer modelo de colocación de malla mosquitero en el cielo raso de la vivienda. Este modelo servirá de guía para dar las pautas de renovación en las viviendas que aceptaron implementar el piloto. Se colectará información sobre la cantidad de malla utilizada en la renovación según el tipo de vivienda, el modelo de renovación según tipo de viviendas, así como los costos relacionados.

Un equipo de biólogos entrenados y especializados hará el conteo de vectores dentro de la vivienda seleccionada (intra domiciliario), en el perímetro de la vivienda y a más de 100 metros de la comunidad (extra domiciliario), antes y al final de la renovación de la vivienda, así como a los tres meses, seis meses y nueve meses.

En la linea de base se evaluarán todas las características físicas y sociodemográficas de las viviendas. En cada control y en el basal se medirán como desenlace de interés de densidad de mosquitos Anopheles. En cada ocasión se recolectarán especímenes de mosquitos de una casa diferente cada noche durante dos noches al mes mediante la ténica de captura humana de mosquitos (*human landing catching*) durante 12 h (18.00 a 06.00), con participación de cuatro colectores y un supervisor por vivienda, y utilizando un protocolo idéntico en exteriores (peridoméstico, dentro de ~ 10 m de cada casa) y en interiores, siguiendo el protocolo descrito por Rosas-Aguirre M et al.[2]

La colecta de vectores se hará en las viviendas casos y controles, para medir posteriormente la densidad, el índice de picadura hombre-noche, el índice de picadura hombre – hora y el nivel de riesgo de transmisión. Así mismo se medirán las condiciones de temperatura y humedad relativa durante la noche y el día dentro de la vivienda, fuera de la vivienda y lejos de la vivienda. En los cuatro periodos establecidos.

Todos los vectores colectados serán llevados en frascos al Laboratorio de Referencia Regional de Loreto, para el análisis de las especies de vectores encontrados y el conteo. Toda la información es vaciada al Formato de Vigilancia Entomológica. Ver anexo.

De forma adicional, un equipo de encuestadores aplicará la Encuesta Familiar en todas las viviendas de la comunidad para determinar los horarios y número de horas que pasa dentro de la vivienda y dentro del mosquitero impregnado con insecticida de larga duración en los días de semana y fin de semana.

Finalmente, la IPRESS Ninarumi estará a cargo de realizar el diagnóstico de malaria en la comunidad a través de la prueba de gota gruesa, siguiendo la programación regular de su intervención ya sea a través de la búsqueda activa de casos de malaria, barrido hemático o atenciones intramurales a demanda. Así los casos diagnosticados recibirán el tratamiento que corresponda según las normativas de salud vigentes.

Esto con la finalidad de que el estudio no interfiera en las actividades regulares que realiza la IPRESS en el diagnóstico y tratamiento de la malaria en la comunidad de Llanchama. Ya que un diagnostico masivo e intencional por razones del estudio podría conllevar también a una administración masiva de tratamiento, por lo que la inclusión de este factor en el estudio dificultaría la observación del efecto de renovación de viviendas con malla mosquitero en la tasa de incidencia de malaria.

La información secundaria que provea la IPRESS de Ninarumi sobre el diagnostico de casos de malaria en la comunidad de Llanchama, será utilizada para determinar la tasa de incidencia acumulada; antes, después de la renovación, aproximadamente a los seis meses y al año del estudio.

4.6 Procesamiento de información

La información de la cantidad de malla mosquitero y otros materiales utilizados, los costos de la renovación de viviendas según tipo, la información del formato de vigilancia entomológica, los resultados del test de gota gruesa y la encuesta, familiar serán doble digitados con filtros para categorías y rangos para evitar errores de digitación utilizando Microsoft Excel. Luego se realizará un análisis descriptivo resumiendo las variables cualitativas según su frecuencia absoluta y relativa, las variables cuantitativas de distribución normal según su media y desviación normal, y las variables cuantitativas de distribución no normal según su mediana y rango intercuartílico. Adicionalmente se realizará un análisis gráfico con la distribución espacial de los conteos de mosquitos por casa con el objeto de identificar potenciales conglomerados. Finalmente, para determinar si la colocación de la malla mosquitero tiene un efecto en los conteos de mosquitos capturados en el intra-domiciliario y en el extra-domiciliario se ajustarán modelos de regresión binomial negativo multinivel ajustando el efecto estimado por los confusores conocidos. En este modelamiento se considerarán como confusores a las siguientes variables independientes: secuencia, casa, lugar de la picadura (exofágica/endofágica), período de tiempo (18.00 a 21.00, 21.00 a 00.00, 00.00 a 03.00, 03.00 a 06.00). Para ello usaremos el software R v4.3.1 y la función glm.nb() del paquete MASS. Adicionalmente se explorarán interacciones de segundo y tercer orden utilizando el método forward de modelos anidados.

4.7 Socialización de resultados

Los resultados serán socializados con actores claves de salud a nivel nacional, en la región de Loreto, así como autoridades locales de los distritos de San Juan Bautista y otros aledaños que tengan interés en esta información.

El análisis y publicación de los resultados se realizará manteniendo el anonimato de los participantes.

4.8 Propiedad intelectual

La propiedad intelectual del estudio le pertenecerá a la Gerencia Regional de Salud de Loreto, World Vision y KOICA.

5 Consideraciones éticas

Toda la información será recolectada posterior a la aprobación del Comité de Ética del Hospital Regional de Loreto.

El consentimiento informado será firmado por el jefe de familia de cada vivienda en señal de aceptación de participar él y su familia en el estudio. Tanto niños, niñas, adolescentes,

como sus padres, madres o cuidadores serán informados sobre los objetivos del estudio y el procedimiento de recolección de información.

Así mismo, se les informará que los resultados de la prueba de gota gruesa realizada e información del tratamiento de malaria que hayan recibido por la IPRESS de su jurisdicción serán utilizados para conocer la incidencia de la malaria en su comunidad.

También los especialistas involucrados en la aplicación de la técnica de cebo humano están debidamente capacitados y certificados para reducir al riesgo de contraer malaria durante el recojo de información en este estudio y de aceptar su participación en este estudio realizando la coleta de vectores firmarán un documento de consentimiento informado sobre los riesgos, como señal de conformidad y aceptación de su participación

6 Presupuesto

El presupuesto disponible está estimado durante el año que dura el estudio. Cabe precisar que los costos son asumidos entre la Gerencia Regional de Salud de Loreto y World Vision Perú a través del proyecto financiado por KOICA. Algunos de estos costos son compartidos en efectivo, en recursos humanos y en especies.

DESCRIPCIÓN	MONTO ESTIMADO (S/.)	FINANCIADO POR
Materiales para la renovación de viviendas con malla mosquitero	21,600.00	Proyecto RTA
Servicio de analista estadístico y equipo en campo especializado en diseños experimentales	7,000.00	Proyecto RTA
Servicio de revisión por el comité de ética	580.00	Proyecto RTA
Actividades regulares de búsqueda activa de malaria en la comunidad	2500.00	GERESA
Servicio de entomología	3,000.00	GERESA
Materiales de papelería y otros relacionados a salud	1000.00	Proyecto RTA
Entrenamiento de personal	3000.00	GERESA
Alimentación y transporte de personal	7000.00	Proyecto RTA
Servicio de colecta de vectores por equipo especializado	60,000.00	Proyecto RTA (57%) GERESA (43%)
Ceremonia de socialización de resultados	5,000.00	
**TOTAL**	**110,680.00**	

7 Cronograma

	J	F	M	A	M	J	J	A	S	O	N
Elaboración del Diseño del estudio	X										
Aprobación del comité de ética	X										
Capacitación al personal	X										
Adquisición de equipos e insumos	X		X			X		X			
Medición basal	X	X									
Control 1				X	X						
Control 2							X	X			
Control 3									X	X	
Análisis de los resultados		X			X			X		X	
Presentación del informe final											X
Evento de socialización de resultados											X

8 Anexos

- Consentimiento informado e instructivo del estudio
- Formato de vigilancia entomológica
- Lista de materiales y costos para renovación de viviendas
- Encuesta Familiar – Horarios de permanencia dentro de vivienda y mosquitero
- Consentimientos firmados por especialistas para la técnica de cebo humano

9 Referencias bibliográficas

1. Roshanravan B, Kari E, Gilman RH, Cabrera L, Lee E, Metcalfe J, Calderon M, Lescano AG, Montenegro SH, Calampa C, Vinetz JM, 2003. Endemic malaria in the Peruvian Amazon region of Iquitos. Am J Trop Med Hyg 69: 45-52.
2. Rosas-Aguirre A, Guzman-Guzman M, Moreno-Gutierrez D, Rodriguez-Ferrucci H, Vargas-Pacherrez D, Acuna-Gonzalez Y, 2011. [Long-lasting insecticide - treated bednet ownership, retention and usage one year after their distribution in Loreto, Peru]. Rev Peru Med Exp Salud Publica 28: 228-36.

ANEXO 1: CONSENTIMIENTO INFORMADO CON INSTRUCTIVO

CONSENTIMIENTO INFORMADO

Nombre del estudio:

ESTUDIO EXPERIMENTAL DE DENSIDAD DE VECTORES EN VIVIENDAS RENOVADAS CON MALLA MOSQUITERO, COMUNIDAD LLANCHAMA, DISTRITO DE SAN JUAN BAUTISTA, PROVINCIA DE MAYNAS, REGIÓN LORETO

¿De qué trata este estudio?

La Gerencia Regional de Salud de Loreto, la ONG World Vision Perú y KOICA actualmente vienen implementando en asocio el Proyecto "Reducción de la Carga de Enfermedades Tropicales Desatendidas y promoción de la Salud Básica en la Amazonía Peruana" con la finalidad de contribuir en la reducción de los casos de dengue y malaria en el distrito de San Juan Bautista.

Actualmente estamos próximos a implementar el piloto de renovación de viviendas, colocando malla mosquitero para reducir la cantidad de vectores dentro de la vivienda y por consiguiente esperamos reducir los casos de malaria en su comunidad.

Esta es la razón del estudio y su colaboración nos permitirán saber si esta estrategia es efectiva y posteriormente en caso de serlo, podremos difundirla en toda la región, para que otras familias la conozcan y puedan protegerse de estas enfermedades.

Por favor, lea detenidamente este documento, haga todas las preguntas que crea pertinentes antes de decidir su participación en el estudio. Le explicaremos los detalles del estudio y le dejaremos una copia de este formato de consentimiento para que lo conserve.

¿Qué va a ocurrir durante el estudio?

Este estudio se desarrollará tanto en viviendas que decidieron renovar su casa colocando malla mosquitero y también en aquellas que no han decidido hacerlo dentro de la comunidad de Llanchama.

Si usted está de acuerdo en participar en el estudio haremos lo siguiente:

- Le solicitaremos su consentimiento, el cual debe firmarlo.
- Un equipo de expertos pasará durante toda una noche, colectando zancudos, midiendo la temperatura y la humedad. El horario usualmente será entre 6 p.m. a 6 a.m.
- Los expertos colectarán los zancudos y medirán la temperatura y humedad cada hora dentro de su vivienda, también lo harán fuera de su vivienda y a 100 metros fuera de la comunidad.
- Esta recolección de zancudos y medición de temperatura y humedad se hará antes de que en su comunidad se empiece la renovación de las viviendas, inmediatamente después de terminada la renovación, y se repetirá a los seis meses y al año, en total serán cuatro veces, durante todo un año.
- Así mismo, recibirá la visita de encuestadores quienes pedirán que el jefe de familia, conteste algunas preguntas que nos ayudarán a completar el estudio.
- Así mismo, si durante el año, usted o algún miembro de su familia se ha realizado alguna prueba para descartar malaria, pedimos su autorización para revisar esta información y analizar cuál es la situación de la malaria en su comunidad.

- El resultado del tipo y número de zancudos encontrados dentro de su vivienda, se le hará llegar a su domicilio para que pueda conocerlos y tomar las medidas que sean necesarias para proteger a su familia.

¿Tiene algún beneficio participar en el estudio?

Los beneficios de participar en este estudio es que podrá conocer la cantidad de zancudos que hay en su vivienda y usted y su familia podrá tomar medidas preventivas para evitar enfermarse.

Si usted decide participar o no, no tiene ninguna influencia o es una condición para participar en otras actividades que la GERESA Loreto, World Vision y KOICA estén realizando en su comunidad.

¿Participar en el estudio me hará daño?

El ruido que pudiera haber durante la colecta de zancudos dentro de su vivienda podría generarle alguna incomodidad mientras su familia duerme, pero ningún daño.

¿Cómo será protegida mi privacidad?

Su privacidad será protegida porque los datos serán manejados con códigos, no con su nombre ni el de su familia. Los resultados serán confidenciales solo los investigadores involucrados en este estudio tendrán acceso a la misma.

Después de leer esta hoja de información, he tenido tiempo suficiente para considerar mi decisión. Me han dado la oportunidad de formular preguntas y todas ellas se respondieron satisfactoriamente.

Comprendo que la participación es voluntaria y que puedo retirarme del estudio:

- Cuando quiera.
- Sin tener que dar explicaciones.
- Sin que esto repercuta en mis cuidados médicos.

Después de haber meditado sobre la información que me han proporcionado, declaro que:

Yo...

(Nombre y apellidos del jefe de familia)

Con DNI Nª: ………………………………………..manifiesto que mi decisión es la siguiente:

☐ Doy ☐ No doy

Mi consentimiento para el acceso y utilización de mis datos y de mi familia en las condiciones detalladas en la hoja de información.

FIRMA DEL JEFE DE FAMILIA	FIRMA DE LA PERSONA QUE ADMINISTRÓ EL CONSENTIMIENTO
NOMBRE:	NOMBRE:
FECHA:	FECHA:

ANEXO 2: FORMATO DE VIGILANCIA ENTOMOLÓGICA

FICHA DE COLECTA DE MOSQUITOS ADULTOS

UBICACIÓN GEOGRAFICA		DATOS COMPLEMENTARIOS
GERESA: LORETO	RED/MICRORED:____________________	ALTITUD DE LA LOCALIDAD: ___________________
PROVINCIA:_____________________	ESTABLEC. DE SALUD: ______________________________	PG. Lat. Sur:__________________
DISTRITO:______________________	VALLE/QUEBRADA: _______________________	Long Oeste: _________________
LOCALIDAD: ____________________	RIO/AFLUENTE: _______________________	

TIPO DE VIVIENDA: A ( ) B ( ) C ( ) D ( )

DIRECCIÓN Y NOMBRE DEL JEFE DE FAMILIA:________________________________

MÉTODO DE COLECTA:________________________________

FECHA DE COLECTA:_________________

DISTANCIA AL CRIADERO MÁS CERCANO (mts.):_____________

FECHA ULTIMO TRATAMIENTO: __________________

TIPO DE TRATAMIENTO: Residual ( ) Espacial ( )

INSECTICIDA UTILIZADO:_________________________________

HORA	HR %	Temp °C	COLECTA MOSQUITOS.				DETERMINACION DE ESPECIES																															OBSERVACIONES (Factores climatológicos y otras eventualidades)
							INTRA										PERI										EXTRA										
							Especies Identificadas por el laboratorio						Disección Ovarios			Otros culicideos	Especies Identificadas por el laboratorio						Disección Ovarios			Otros culicideos	Especies Identificadas por el laboratorio						Disección Ovarios			Otros culicideos	
			Intra	Peri	Extra	Total							Nul.	Par.	Total								Nul.	Par.	Total								Nul.	Par.	Total		
18:00 - 19:00																																					
19:00 - 20:00																																					
20:00 - 21:00																																					
21:00 - 22:00																																					
22:00 - 23:00																																					
23:00 - 00:00																																					
00:00 - 01:00																																					
01:00 - 02:00																																					
02:00 - 03:00																																					
03:00 - 04:00																																					
04:00 - 05:00																																					
05:00 - 06:00																																					
TOTAL																																					

COLECTORES: ____

____
Nombre y Firma del Colector Responsable

IPHH:__________

IPHH Peri:__________

IPHH Intra:__________

IPHN: ___________

IPHN Peri: ___________

IPHN Intra: ___________

ANEXO 3: LISTA DE MATERIALES Y COSTOS PARA RENOVACIÓN DE VIVIENDAS

I. DATOS GENERALES DE LA VIVIENDA

**NOMBRE DEL JEFE DE FAMILIA**			
**DIRECCIÓN:**			
**DIMENSIONES DE LA VIVIENDA (Metros)**	**Frente: (metros) Fondo : (metros)**		
**TIPO DE MATERIALES EN PAREDES**		**TIPO DE MATERIAL EN TECHO**	
**TIPO DE VIVIENDA**		**TIPO DE SSHH**	
**Nª HABITACIONES**		**FECHA:**	

II. MATERIALES Y COSTOS DE LA VIVIENDA

MATERIALES Y SERVICIOS	UNID.	CANT.	COSTO UNITARIO (S/.)	COSTO TOTAL (S/.)
**Servicio de colocación de malla mosquitero**	M2			
**Servicio de instalación de sistema eléctrico**	Metro lineal			
**Servicio de colocación de puntos eléctricos**	X punto			
**Costo listones de madera**				
LISTONES DE MADERA 3X2X4	Unidad			
LISTON DE MADERA 4X2X4 M	Unidad			
LISTON DE MADERA 2X1X4 M	Unidad			
LISTON DE MADERA 2X2X4 M	Unidad			
LISTON DE MADERA 4X4X4 M	Unidad			
**Costo clavos**				
CLAVO 2 1/2	Kilo			
CLAVO DE 3 PULG PARA MADE	Kilo			
CLAVO DE 2 PARA MADERA	Kilo			
CLAVO DE 3 PULG PARA MADE	Kilo			
CLAVO 4 PARA MADERA x Kg	Kilo			
CLAVO PARA MADERA 1 1/2	Kilo			
CLAVO 4 PARA MADERA x Kg	Kilo			
CLAVO DE 1" PARA MADERA	Kilo			
**Costo de malla mosquitero**				
MALLA MOSQUITERO	Metro			
**Otros costes**				
TOTAL				

ANEXO 4: HORARIO DE PERMANENCIA EN LA VIVIENDA Y DENTRO DEL MOSQUITERO

I. DATOS GENERALES DE LA FAMILIA

**NOMBRE DEL JEFE DE FAMILIA**			
**DIRECCIÓN:**			
**EN QUÉ TRABAJA**			
**CUANTAS PERSONAS VIVEN AQUÍ**		**ALGUIEN QUE VIVE AQUÍ, TUVO ALGUNA VEZ MALARIA (si/no)**	
**HAY UN PUNTO DONDE SE ACUMULA EL AGUA CERCA A SU CASA (menos de 50 m)**		**TIPO DE PUNTO DE AGUA**	
**QUE USA PARA EVITAR LA PICADURA DEL ZANCUDO**		**ULTIMA FECHA EN QUE FUMIGARON SU CASA**	

III. USO MOSQUITERO TRATADO CON INSECTICIDA DE LARGA DURACIÓN

Anote los nombres y edad de todos los miembros de la familia en el recuadro y responda SI o NO durmió bajo mosquitero **el día viernes.**

Nombres							
A que se dedica							
Edad							
Con mosquitero (MTILD) SI/NO							
Otro tipo de mosquitero SI/NO							

Nombres							
A que se dedica							
Edad							
Con mosquitero (MTILD) SI/NO							
Otro mosquitero SI/NO							

LORETO GERESA GERENCIA REGIONAL DE SALUD LORETO

KOICA Korea International Cooperation Agency

World Vision

IV. HORARIO EN EL QUE PERMANECE DENTRO DE CASA Y DENTRO DEL MOSQUITERO DE LUNES A VIERNES

Instrucciones: Colocar el primer nombre de cada persona que vive en la vivienda. Luego marcar con una X en la columna de la letra "C" según las horas que **el último viernes permaneció** dentro de casa (de la puerta principal hacia adentro). Si estuvo tomando aire en la puerta de su casa, esto se considera fuera de casa. Marcar en la columna "M" en el horario que permaneció dentro del mosquitero **el último viernes.**

Horario	Nombres de los miembros de la familia																			
	C	M	C	M	C	M	C	M	C	M	C	M	C	M	C	M	C	M	C	M
4 - 5 am																				
5 - 6 am																				
6 - 7 am																				
7 - 8 am																				
8 - 9 am																				
9 -10 am																				
10 - 11 am																				
11 - 12 pm																				
1 - 2 pm																				
2 - 3 pm																				
3 - 4 pm																				
4 - 5 pm																				
5 - 6 pm																				
6 - 7 pm																				
7 - 8 pm																				
8 - 9 pm																				
9-10 pm																				
10 - 11 pm																				
11 - 12 am																				
12 – 4 am																				

OBSERVACIONES

V. HORARIO EN EL QUE PERMANECE DENTRO DE CASA Y DENTRO DEL MOSQUITERO LOS SÁBADOS Y DOMINGOS

Instrucciones: Colocar el primer nombre de cada persona que vive en la vivienda. Luego marcar con una X en la columna de la letra “C” según las horas que **ayer permaneció** dentro de casa (de la puerta principal hacia adentro). Si estuvo tomando aire en la puerta de su casa, esto se considera fuera de casa. Marcar en la columna “M” en el horario que permaneció dentro del mosquitero **el día de ayer**.

Horario	Nombres de los miembros de la familia																			
	C	M	C	M	C	M	C	M	C	M	C	M	C	M	C	M	C	M	C	M
4 - 5 am																				
5 - 6 am																				
6 - 7 am																				
7 - 8 am																				
8 - 9 am																				
9-10 am																				
10 - 11 am																				
11 - 12 pm																				
1 - 2 pm																				
2 - 3 pm																				
3 - 4 pm																				
4 - 5 pm																				
5 - 6 pm																				
6 - 7 pm																				
7 - 8 pm																				
8 - 9 pm																				
9-10 pm																				
10 - 11 pm																				
11 - 12 am																				
12 – 4 am																				
OBSERVACIONES																				

ANEXO 5: CONSENTIMIENTO INFORMADO PARA ESPECIALISTAS DE TÉCNICA DE CEBO HUMANO

CONSENTIMIENTO INFORMADO

Nombre del estudio:

ESTUDIO EXPERIMENTAL DE DENSIDAD DE VECTORES EN VIVIENDAS RENOVADAS CON MALLA MOSQUITERO, COMUNIDAD LLANCHAMA, DISTRITO DE SAN JUAN BAUTISTA, PROVINCIA DE MAYNAS, REGIÓN LORETO

¿De qué trata este estudio?

La Gerencia Regional de Salud de Loreto, la ONG World Vision Perú y KOICA actualmente vienen implementando en asocio el Proyecto "Reducción de la Carga de Enfermedades Tropicales Desatendidas y promoción de la Salud Básica en la Amazonía Peruana" con la finalidad de contribuir en la reducción de los casos de dengue y malaria en el distrito de San Juan Bautista.

Actualmente estamos próximos a implementar el piloto de renovación de viviendas, colocando malla mosquitero para reducir la cantidad de vectores dentro de la vivienda y por consiguiente esperamos reducir los casos de malaria en la comunidad de Llanchama.

Esta es la razón del estudio y su colaboración nos permitirán saber si esta estrategia es efectiva y posteriormente en caso de serlo, podremos difundirla en toda la región, y mejorar las políticas públicas que contribuyan en la reducción de la malaria.

Por favor, lea detenidamente este documento, haga todas las preguntas que crea pertinentes antes de decidir su participación en el estudio. Le explicaremos los detalles del estudio y le dejaremos una copia de este formato de consentimiento para que lo conserve.

¿Qué va a ocurrir durante el estudio?

Este estudio se desarrollará tanto en viviendas que decidieron renovar su casa colocando malla mosquitero y también en aquellas que no han decidido hacerlo dentro de la comunidad de Llanchama. La medición se realizará antes, después, a los seis meses y al año de la renovación.

Usted como Especialista en la técnica de cebo humano, realizará el conteo de vectores hematófagos entre 6 p.m. a 6 a.m., en el área intradomiciliario, peri domiciliario y extradomiciliario, luego la información recolectada será entregada a su supervisor de la Dirección de Salud Ambiental de la Gerencia Regional de Salud de Loreto, para que realicen el cálculo de todos los indicadores de riesgo de transmisión de malaria.

Esta información y otras que serán recopiladas en la comunidad ayudarán a determinar los hallazgos relacionados a los objetivos de este estudio.

Si usted está de acuerdo en asumir este rol durante el estudio, deberá firmar este consentimiento informado y presentar un certificado de su ultima capacitación, la cual deberá ser menor a un año.

¿Tiene algún beneficio participar en el estudio?

Además de la experiencia profesional que pueda proveerle participar en este estudio experimental, como parte del asocio entre GERESA Loreto, World Vision y KOICA, su participación y su rol serán mencionados en los créditos del estudio.

¿Participar en el estudio tendrá algún riesgo?

Como es de su conocimiento, aplicar la técnica del cebo humano puede colocarle en riesgo de contraer malaria, sin embargo, este riesgo se reduce en tanto usted aplique la técnica de forma correcta e informe a su supervisor cualquier imprevisto e incidente sobre el que no pueda tener control y que ponga en riesgo su salud, para tomar cualquier medida preventiva oportunamente.

Así mismo por su seguridad, estará acompañado todo el tiempo de otra persona del equipo de la Dirección de Salud Ambiental u otra de la comunidad quién le dará soporte mientras ejecuta la técnica y se asegurará que usted no corra ningún peligro si tuviese que hacer el conteo en el intradomicilio, peridomicilio y extradomicilio.

Después de haber meditado sobre la información que me han proporcionado, declaro que:

Yo...

(Nombre y apellidos)

Con DNI Nª: ....manifiesto que mi decisión es la siguiente:

☐ Doy ☐ No doy

Mi consentimiento para realizar la técnica de cebo humano, según las condiciones detalladas en la hoja de información.

FIRMA DEL ESPECIALISTA EN TÉCNICA DE CEBO HUMANO	FIRMA DE LA PERSONA QUE ADMINISTRÓ EL CONSENTIMIENTO
NOMBRE:	NOMBRE:
FECHA:	FECHA:

Translated from Spanish to English - www.onlinedoctranslator.com

EXPERIMENTAL STUDY OF VECTOR DENSITY IN RENOVATED HOUSES WITH MOSQUITO NET MESH, LLANCHAMA COMMUNITY, SAN JUAN BAUTISTA DISTRICT, MAYNAS PROVINCE, LORETO REGION

Produced by:

World Vision Peru

Loreto Regional Health Management

KOICA

SEPTEMBER 2023

Content

1 Background 3

2 Project proposal 3

3 The purpose of the study 4

3.1 General objective 4

3.2 Specific objectives 4

4 Methodology 4

4.1 Study design 4

4.2 Population and sample 4

4.3 Sampling technique 5

4.4 Exclusion criteria 5

4.5 Information collection procedure 5

4.6 Information processing and analysis ..... **¡Error! Marcador no definido.**

4.7 Socialization of results 7

4.8 Intellectual property 7

5 Ethical considerations 7

6 Budget 7

7 Schedule 8

8 Annexes 8

1 Background

Despite the reduction in mortality from Malaria in the world by 60% during the period 2000 to 2019, it is still very high and unacceptable. Until April 2023 in the Peruvian Amazon, in the department of Loreto, the number of malaria cases was 11,163.

Since 2018, the Malaria Elimination Plan, called the Zero Malaria Plan (PMC), has been implemented with a validity until 2021 and until 2020, it had managed to reduce cases by 75% in the Loreto region and has prevented more than 80 thousand cases in this period.

However, due to the COVID-19 pandemic, some activities were discontinued or decreased. Therefore, currently, actions for the elimination of malaria are integrated into the Multisectoral Strategic Plan for 2030 of the National Multisectoral Health Policy "Peru, a Healthy Country", with the objective of reducing malaria by 90% in 2030 in Peru.

Among the alternative solutions proposed by the government and that are being implemented in the department of Loreto include: identification of cases through passive detection and active search for cases, detection and diagnosis of cases within 24 hours of the onset of the condition. clinical, training of community agents and health workers, epidemiological surveillance, reducing the risk of contact between man and the vector through the use of insecticide-treated mosquito nets (ITN), application of intra-domiciliary residual spraying, environmental planning, intergovernmental coordination , intersectoral and community participation, development of a communication plan and monitoring incorporating an Ad hoc team at the regional level.

All these actions are part of the four components (early diagnosis, timely treatment, research and response) of the Plan's strategy towards the Elimination of Malaria by 2030.

2 Project proposal

This study is carried out within the framework of the Project "Reducing the Burden of Neglected Tropical Diseases and promoting Basic Health in the Peruvian Amazon" implemented by the NGO World Vision Peru, in association with KOICA, for the period 2022-2024, in the jurisdiction of the district of San Juan Bautista, province of Maynas, Loreto region. Therefore, the findings of this study will allow us to confirm whether the strategy of renovating homes with mosquito netting allows us to reduce the risk of contact between man and vector and thus avoid cases of malaria.

The project's goal hopes to contribute to the reduction of the incidence rate of dengue and malaria in the district of San Juan Bautista, to achieve this it has the following key actions:

- Strengthen the health system: through the development of capabilities in health personnel, implementation of supplies and equipment for the early diagnosis of malaria and dengue, improve the quality of epidemiological information by accessing the internet service through radio installation links and expand the health service to the community for the active search for cases through itinerant health campaigns.
- Influences on behavioral change in the community, so that they adopt preventive measures to avoid dengue and malaria through capacity development for community health agents, dissemination of preventive messages using mass media, contributing to the coverage of fumigated homes. According to prioritized communities, promote prevention measures in schools and test the housing

renovation strategy using mosquito netting as a malaria prevention measure.

The latter is the reason for this study and it is expected to use its findings for local, regional, and/or national incidence and health promotion in the community.

3 The purpose of the study

3.1 General objective

- Determine if the renovation of homes with mosquito netting has an effect on the reduction of the Anopheles vector inside the home.

3.2 Specific objectives

- Determine the effect of renovating homes with the placement of mosquito nets on the ceiling of the home on the reduction of the Anopheles vector inside the home.
- Determine the effect of renovating homes with the placement of mosquito nets on the ceiling of the home on the reduction of the Anopheles vector outside the home.
- Determine the guidelines for the renovation of homes according to their types, placing mosquito netting as a factor to reduce the density of the Anopheles vector and other hematophages.
- Determine the level of risk of malaria transmission according to indicators of the Anopheles vector and other hematophages.
- Determine the cumulative incidence rate of malaria through the measurement of the Annual Parasite Index (API).
- Determine the temperature and relative humidity inside, outside and away from the home.
- Determine the schedules and number of hours family members stay inside and outside the home.
- Determine schedules and number of hours that family members stay inside the mosquito net treated with long-lasting insecticide.

4 Methodology

4.1 Study design

This study will follow a randomized experimental design of stepped groups (stepped wedge cluster randomized trial) with the objective of evaluating the effect of placing mosquito nets on the ceiling of homes on the decrease in the density of the Anopheles vector. , in the community of Llanchama, district of San Juan Bautista, Loreto, Peru.

Malaria in this district has an unstable epidemiological pattern and seasonal behavior, with a peak between the months of March to August. All age groups are affected and most cases are symptomatic. The main vector of malaria is Anopheles darlingi, but the circulation of Anopheles benarrochi has also been reported in the region.[1]For this reason, the study is expected to be carried out over a period of one year and will include a baseline evaluation and four quarterly escalations.

4.2 Population and sample

The study population will be made up of the entire Llanchama community, which has a total of 85 homes with permanent residents. These will be intervened in a phased manner at a rate of 19 houses, which will be taken at random until the intervention is completed in no less than 67% of the houses in the community (Graph 1).

Figure 1. Stepped sequence of the study

Sequence	Basal	Control 1	Control 2	Control 3
Sequence 1	19	19	19	19
Sequence 2	19	19	19	19
Sequence 3	19	19	19	19
Total Housing	57 (57 vs 0)	57 (38 vs 19)	57 (19 vs 38)	57 (0 vs 57)

* White, Control; Grey, Intervention

As the objective of controlling by type of house, the sampling will be weighted in order to enroll the same proportion of houses of types A, B, C and D in each sequence of the study and try to represent the distribution of houses according to type of housing in the community, which are distributed at a rate of approximately 10%, 20%, 30% and 40%.

Assuming a study power of 80%, an alpha of 0.05, an expected effect of a 50% reduction in the count of Anopheles darlingi biting inside houses, it was estimated that a minimum of 57 houses were required in a randomized experimental design of staggered groups (at a rate of 19 houses per step) with a baseline and three controls.

4.3 Sampling and enrollment technique

Taking into consideration the characteristics of the homes of the cases and controls and their types, according to the categorization made by the Directorate of Environmental Health, they will be identified according to the list of homes in the Llanchama community, which will be previously georeferenced and will be listed based on their locations on the community map.

Each head of family in the selected homes will be informed about the study and if they do not agree to participate for any reason, they will be replaced at random by another home that has the same characteristics.

Each family will sign an informed consent in advance and will access a document and detailed explanation about the collection of vectors using the human bait technique and other scope of the study. Likewise, during the process you can answer any questions and you will be free to change your mind and withdraw from the study at any time.

4.4 Exclusion criteria

All homes that have been misted or received residual indoor spraying in the last 90 days will be excluded from the study.

Also those homes in the case group that have not completely renovated their home will be excluded from the study.

4.5 Information collection procedure

Based on the experience of the home renovation pilot in 2022, the first model for placing mosquito netting on the ceiling of the home has been established. This model will serve as a guide to provide renovation guidelines in the homes that agreed to implement the pilot. Information will be collected on the amount of mesh used in the renovation according to the type of home, the renovation model according to the type of home, as well as the related costs.

A team of trained and specialized biologists will count the vectors inside the selected home (intra-domiciliary), on the perimeter of the home and more than 100 meters from

the community (extra-domiciliary), before and at the end of the renovation of the house. housing, as well as at three months, six months and nine months.

In the baseline, all the physical and sociodemographic characteristics of the homes will be evaluated. In each control and at baseline, Anopheles mosquito density will be measured as an outcome of interest. On each occasion, mosquito specimens will be collected from a different house every night for two nights a month using the human landing catching technique for 12 hours (6:00 p.m. to 6:00 a.m.), with the participation of four collectors and a supervisor per housing, and using an identical protocol outdoors (peridomestic, within ~ 10 m of each house) and indoors, following the protocol described by Rosas-Aguirre M et al.[2]

The collection of vectors will be done in the case and control homes, to subsequently measure the density, the man-night bite index, the man-hour bite index and the level of transmission risk. Likewise, temperature and relative humidity conditions will be measured during the night and day inside the home, outside the home and away from the home. In the four established periods.

All the vectors collected will be taken in bottles to the Regional Reference Laboratory of Loreto, for the analysis of the vector species found and the counting. All information is entered into the Entomological Surveillance Form. See Annex.

Additionally, a team of interviewers will apply the Family Survey in all homes in the community to determine the hours and number of hours spent inside the home and inside the mosquito net impregnated with long-lasting insecticide on weekdays and weekends. of week.

Finally, the IPRESS Ninarumi will be in charge of carrying out the diagnosis of malaria in the community through the thick smear test, following the regular programming of its intervention either through the active search for malaria cases, blood sweep or medical care. intramurals on demand. Thus, the diagnosed cases will receive the corresponding treatment according to current health regulations.

This is so that the study does not interfere with the regular activities carried out by the IPRESS in the diagnosis and treatment of malaria in the community of Llanchama. Since a massive and intentional diagnosis for reasons of the study could also lead to a massive administration of treatment, so the inclusion of this factor in the study would make it difficult to observe the effect of renovation of homes with mosquito netting on the incidence rate of malaria.

The secondary information provided by the Ninarumi IPRESS on the diagnosis of malaria cases in the Llanchama community will be used to determine the cumulative incidence rate; before, after the renewal, approximately six months and one year after the study.

4.6 Information processing

The information on the amount of mosquito netting and other materials used, the costs of housing renovation according to type, the information on the entomological surveillance format, the results of the thick blood smear test and the family survey will be double-entered with filters for categories. and ranges to avoid typing errors using Microsoft Excel. Then a descriptive analysis will be carried out summarizing the qualitative variables according to their absolute and relative frequency, the quantitative variables of normal distribution according to their mean and normal deviation, and the quantitative variables of non-normal distribution according to their median and interquartile range. Additionally, a graphic analysis will be carried out with the spatial distribution of mosquito

counts per house in order to identify potential clusters. Finally, to determine if the placement of the mosquito net has an effect on the counts of mosquitoes captured indoors and outdoors, multilevel negative binomial regression models will be fitted, adjusting the estimated effect for the known confounders. In this modeling, the following independent variables will be considered as confounders: sequence, house, place of bite (exophageal/endophagic), time period (18:00 to 21:00, 21:00 to 00:00, 00:00 to 03:00, 03:00 to 06:00). To do this we will use the R v4.3.1 software and the glm.nb() function from the MASS package. Additionally, second and third order interactions will be explored using the forward method of nested models.

4.7 Socialization of results

The results will be socialized with key health actors at the national level, in the Loreto region, as well as local authorities in the districts of San Juan Bautista and other surrounding areas who are interested in this information.

The analysis and publication of the results will be carried out maintaining the anonymity of the participants.

4.8 Intellectual property

The intellectual property of the study will belong to the Regional Health Management of Loreto, World Vision and KOICA.

5 Ethical considerations

All information will be collected after approval by the Ethics Committee of the Loreto Regional Hospital.

The informed consent will be signed by the head of the family of each household as a sign of acceptance of him and his family participating in the study. Both children, adolescents, and their parents or caregivers will be informed about the objectives of the study and the information collection procedure.

Likewise, they will be informed that the results of the thick smear test performed and information on the malaria treatment they have received from the IPRESS in their jurisdiction will be used to determine the incidence of malaria in their community.

Also, the specialists involved in the application of the human bait technique are duly trained and certified to reduce the risk of contracting malaria during the collection of information in this study and if they accept their participation in this study by carrying out the vector collection, they will sign a document of approval. informed consent about the risks, as a sign of agreement and acceptance of their participation

6 Budget

The available budget is estimated during the year of the study. It should be noted that the costs are assumed between the Regional Health Management of Loreto and World Vision Peru through the project financed by KOICA. Some of these costs are shared in cash, human resources and in kind.

DESCRIPTION	ESTIMATED AMOUNT (S/.)	FINANCED BY
Materials for home renovation with mosquito netting	21,600.00	RTA project

Statistical analyst service and field team specialized in experimental designs	7,000.00	RTA project
Review service by the ethics committee	580.00	RTA project
Regular active malaria screening activities in the community	2500.00	GERESA
Entomology service	3,000.00	GERESA
Stationery and other health-related materials	1000.00	RTA project
Staff training	3000.00	GERESA
Food and transportation of personnel	7000.00	RTA project
Vector collection service by specialized team	60,000.00	RTA project (57%) GERESA (43%)
Results socialization ceremony	5,000.00	
**TOTAL**	**110,680.00**	

7 Schedule

	J.	F	M	TO	M	J.	J.	TO	Yes	EITHER	N
Preparation of the study design	x										
Ethics committee approval	x										
Staff training	x										
Acquisition of equipment and supplies	x		x			x		x			
Basal measurement	x	x									
Control 1				x	x						
Control 2							x	x			
Control 3									x	x	
Analysis of the results		x			x			x		x	
Presentation of the final report											x
Results socialization event											x

8 Annexes

- Informed and instructional consent for the study
- Entomological surveillance format
- List of materials and costs for home renovation
- Family Survey – Hours of stay inside the home and mosquito net
- Consents signed by specialists for the human baiting technique

9 Bibliographic references

1. Roshanravan B, Kari E, Gilman RH, Cabrera L, Lee E, Metcalfe J, Calderon M, Lescano AG, Montenegro SH, Calampa C, Vinetz JM, 2003. Endemic malaria in the Peruvian Amazon region of Iquitos. Am J Trop Med Hyg 69:45-52.
2. Rosas-Aguirre A, Guzman-Guzman M, Moreno-Gutierrez D, Rodriguez-Ferrucci H, Vargas-Pacherrez D, Acuna-Gonzalez Y, 2011. [Long-lasting insecticide - treated bednet

ownership, retention and usage one year after their distribution in Loreto, Peru]. Rev Peru Med Exp Public Health 28: 228-36.

ANNEX 1: INFORMED CONSENT WITH INSTRUCTIONS

INFORMED CONSENT

Study name:

EXPERIMENTAL STUDY OF VECTOR DENSITY IN RENOVATED HOUSES WITH MOSQUITO NET MESH, LLANCHAMA COMMUNITY, SAN JUAN BAUTISTA DISTRICT, MAYNAS PROVINCE, LORETO REGION

What is this study about?

The Regional Health Management of Loreto, the NGO World Vision Peru and KOICA are currently implementing in partnership the Project "Reducing the Burden of Neglected Tropical Diseases and promoting Basic Health in the Peruvian Amazon" with the aim of contributing to the reduction of dengue and malaria cases in the district of San Juan Bautista.

We are currently close to implementing the home renovation pilot, placing mosquito netting to reduce the number of vectors inside the home and therefore we hope to reduce malaria cases in your community.

This is the reason for the study and your collaboration will allow us to know if this strategy is effective and later, if it is, we will be able to spread it throughout the region, so that other families know about it and can protect themselves from these diseases.

Please read this document carefully and ask any questions you think may be relevant before deciding to participate in the study. We will explain the details of the study to you and leave a copy of this consent form for you to keep.

What will happen during the study?

This study will be carried out both in homes that decided to renovate their house by installing mosquito netting and also in those that have not decided to do so within the community of Llanchama.

If you agree to participate in the study we will do the following:

- We will ask for your consent, which you must sign.
- A team of experts will spend an entire night, collecting mosquitoes, measuring temperature and humidity. The hours will usually be between 6 pm to 6 am
- The experts will collect the mosquitoes and measure the temperature and humidity every hour inside your home, they will also do so outside your home and 100 meters outside the community.
- This collection of mosquitoes and measurement of temperature and humidity will be done before the renovation of the homes begins in your community, immediately after the renovation is completed, and will be repeated after six months and a year, a total of four times, for a whole year.
- Likewise, you will receive a visit from interviewers who will ask the head of the family to answer some questions that will help us complete the study.
- Likewise, if during the year, you or a member of your family has undergone a test to rule out malaria, we ask for your authorization to review this information and analyze the malaria situation in your community.

- The result of the type and number of mosquitoes found inside your home will be sent to your home so that you can find out about them and take the necessary measures to protect your family.

Is there any benefit from participating in the study?

The benefits of participating in this study is that you will be able to know the number of mosquitoes in your home and you and your family will be able to take preventive measures to avoid getting sick.

Whether you decide to participate or not does not have any influence or is a condition to participate in other activities that GERESA Loreto, World Vision and KOICA are carrying out in your community.

Will participating in the study hurt me?

The noise that may occur during the collection of mosquitoes inside your home could cause some discomfort while your family sleeps, but no harm.

How will my privacy be protected?

Your privacy will be protected because the data will be handled with codes, not with your name or that of your family. The results will be confidential, only the researchers involved in this study will have access to it.

After reading this information sheet, I have had enough time to consider my decision. I have been given the opportunity to ask questions and all of them have been answered satisfactorily.

I understand that participation is voluntary and that I can withdraw from the study:

- Whenever you want.
- Without having to give explanations.
- Without this affecting my medical care.

After having considered the information provided to me, I declare that:

I...

(Name and surname of the head of the family)

With DNI Nª: .... I declare that my decision is the following:

☐Give☐I do not give

My consent for the access and use of my data and that of my family under the conditions detailed in the information sheet.

SIGNATURE OF THE HEAD OF THE FAMILY	SIGNATURE OF THE PERSON WHO ADMINISTERED THE CONSENT
NAME:	NAME:
DATE:	DATE:

ANNEX 2: ENTOMOLOGICAL SURVEILLANCE FORMAT

FICHA DE COLECTA DE MOSQUITOS ADULTOS

UBICACIÓN GEOGRAFICA		DATOS COMPLEMENTARIOS
GERESA: LORETO	RED/MICRORED:____________________	ALTITUD DE LA LOCALIDAD: ___________________
PROVINCIA:_____________________	ESTABLEC. DE SALUD: ______________________________	PG. Lat. Sur:_________________
DISTRITO:_______________________	VALLE/QUEBRADA: _______________________	Long Oeste: _________________
LOCALIDAD: ____________________	RIO/AFLUENTE: _______________________	

TIPO DE VIVIENDA: A ( ) B ( ) C ( ) D ( )

DIRECCIÓN Y NOMBRE DEL JEFE DE FAMILIA:________________________________

MÉTODO DE COLECTA:__________________________________

FECHA DE COLECTA:_________________

DISTANCIA AL CRIADERO MÁS CERCANO (mts.):_____________

FECHA ULTIMO TRATAMIENTO: __________________

TIPO DE TRATAMIENTO: Residual ( ) Espacial ( )

INSECTICIDA UTILIZADO:________________________________

HORA	HR %	Temp °C	COLECTA MOSQUITOS,				DETERMINACION DE ESPECIES																															OBSERVACIONES (Factores climatológicos y otras eventualidades)
							INTRA										PERI										EXTRA										
			Intra	Peri	Extra	Total	Especies Identificadas por el laboratorio						Disección Ovarios			Otros culicideos	Especies Identificadas por el laboratorio						Disección Ovarios			Otros culicideos	Especies Identificadas por el laboratorio						Disección Ovarios			Otros culicideos	
													Nul.	Par.	Total								Nul.	Par.	Total								Nul.	Par.	Total		
18:00 - 19:00																																					
19:00 - 20:00																																					
20:00 - 21:00																																					
21:00 - 22:00																																					
22:00 - 23:00																																					
23:00 - 00:00																																					
00:00 - 01:00																																					
01:00 - 02:00																																					
02:00 - 03:00																																					
03:00 - 04:00																																					
04:00 - 05:00																																					
05:00 - 06:00																																					
TOTAL																																					

COLECTORES: ____

____
Nombre y Firma del Colector Responsable

IPHH:__________ IPHN: ___________

IPHH Peri:__________ IPHN Peri: ___________

IPHH Intra:__________ IPHN Intra: ___________

ANNEX 3: LIST OF MATERIALS AND COSTS FOR HOUSING RENOVATION

I. GENERAL DATA OF THE HOUSING

**NAME OF THE HEAD OF THE HOUSEHOLD**			
**ADDRESS:**			
**HOUSING DIMENSIONS (Meters)**	**Front: (meters) Depth: (meters)**		
**TYPE OF MATERIALS ON WALLS**		**TYPE OF ROOF MATERIAL**	
**HOUSING TYPE**		**SSHH TYPE**	
**N° ROOMS**		**DATE:**	

II. HOUSING MATERIALS AND COSTS

MATERIALS AND SERVICES	UNIT	QTY	UNIT COST (S/.)	TOTAL COST (S/.)
**Mosquito net installation service**	M2			
**Electrical system installation service**	Lineal meter			
**Electrical point placement service**	X point			
**Cost of wooden slats**				
WOODEN SLATS 3X2X4	Unit			
WOODEN SLAT 4X2X4 M	Unit			
WOODEN SLAT 2X1X4 M	Unit			
WOODEN SLAT 2X2X4 M	Unit			
WOODEN SLAT 4X4X4 M	Unit			
**Nail cost**				
NAIL 2 1/2	Kilo			
3 INCH NAIL FOR MADE	Kilo			
NAIL OF 2 FOR WOOD	Kilo			
3 INCH NAIL FOR MADE	Kilo			
NAIL 4 FOR WOOD x Kg	Kilo			
WOOD NAIL 1 1/2	Kilo			
NAIL 4 FOR WOOD x Kg	Kilo			
1" NAIL FOR WOOD	Kilo			
**Mosquito net cost**				
MOSQUITO NET MESH	Meter			
**Other costs**				
TOTAL				

ANNEX 4: SCHEDULE OF PERMANENCE IN THE HOUSING AND INSIDE THE MOSQUITO NET

I. GENERAL INFORMATION OF THE FAMILY

**NAME OF THE HEAD OF THE HOUSEHOLD**			
**ADDRESS:**			
**WHAT YOU WORK ON**			
**HOW MANY PEOPLE LIVE HERE**		**HAS ANYONE WHO LIVES HERE EVER HAD MALARIA (yes/no)**	
**THERE IS A POINT WHERE WATER ACCUMULATES NEAR YOUR HOUSE (less than 50 m)**		**WATER POINT TYPE**	
**WHAT YOU USE TO PREVENT THE MOSQUITO BITE**		**LAST DATE THEY FUMIGATED YOUR HOUSE**	

III. USE MOSQUITO NET TREATED WITH LONG-LASTING INSECTICIDE

Write the names and ages of all family members in the box and answer YES or NO you slept under a mosquito net on Friday.

Names							
What do you do for a living							
Age							
With mosquito net (LLIN) BUT							
Other type of mosquito net BUT							

Names							
What do you do for a living							
Age							
With mosquito net (LLIN) BUT							
Other mosquito							

net YES/NO							

IV. SCHEDULE IN WHICH YOU STAY INSIDE THE HOUSE AND INSIDE THE MOSQUITO NET FROM MONDAY TO FRIDAY

Instructions: Enter the first name of each person who lives in the home. Then mark with an If you were getting air outside your house, this is considered outside the home. Mark in column "M" the time you spent inside the mosquito net last Friday.

Schedule	Names of family members																			
	c	M	c	M	c	M	c	M	c	M	c	M	c	M	c	M	c	M	c	M
4 - 5am																				
5 - 6am																				
6 - 7am																				
7 - 8am																				
8 - 9am																				
9 -10 am																				
10 - 11am																				
11 - 12 pm																				
1 - 2 p.m.																				
2 - 3 p.m.																				
3 - 4 p.m.																				
4 - 5 p.m.																				
5 - 6 p.m.																				
6 - 7 p.m.																				
7 - 8 p.m.																				
8 - 9 p.m.																				
9-10 p.m.																				
10 - 11 pm																				
11 - 12 am																				
12 – 4am																				

OBSERVATIONS

V. SCHEDULE IN WHICH YOU STAY INSIDE THE HOUSE AND INSIDE THE MOSQUITO NET ON SATURDAYS AND SUNDAYS

GERESA
GERENCIA REGIONAL
DE SALUD LORETO

KOICA
Korea International
Cooperation Agency

World Vision

Instructions: Enter the first name of each person who lives in the home. Then mark with an If you were getting air outside your house, this is considered outside the home. Mark in column “M” the time that you stayed inside the mosquito net yesterday.

Schedule	Names of family members																			
	c	M	c	M	c	M	c	M	c	M	c	M	c	M	c	M	c	M	c	M
4 - 5am																				
5 - 6am																				
6 - 7am																				
7 - 8am																				
8 - 9am																				
9-10am																				
10 - 11am																				
11 - 12 pm																				
1 - 2 p.m.																				
2 - 3 p.m.																				
3 - 4 p.m.																				
4 - 5 p.m.																				
5 - 6 p.m.																				
6 - 7 p.m.																				
7 - 8 p.m.																				
8 - 9 p.m.																				
9-10 p.m.																				
10 - 11 pm																				
11 - 12 am																				
12 – 4am																				
OBSERVATIONS																				

ANNEX 5: INFORMED CONSENT FOR HUMAN BAIT TECHNIQUE SPECIALISTS

INFORMED CONSENT

Study name:

EXPERIMENTAL STUDY OF VECTOR DENSITY IN RENOVATED HOUSES WITH MOSQUITO NET MESH, LLANCHAMA COMMUNITY, SAN JUAN BAUTISTA DISTRICT, MAYNAS PROVINCE, LORETO REGION

What is this study about?

The Regional Health Management of Loreto, the NGO World Vision Peru and KOICA are currently implementing in partnership the Project "Reducing the Burden of Neglected Tropical Diseases and promoting Basic Health in the Peruvian Amazon" with the aim of contributing to the reduction of dengue and malaria cases in the district of San Juan Bautista.

We are currently close to implementing the housing renovation pilot, placing mosquito netting to reduce the number of vectors inside the home and consequently we hope to reduce malaria cases in the Llanchama community.

This is the reason for the study and your collaboration will allow us to know if this strategy is effective and later, if it is, we will be able to disseminate it throughout the region, and improve public policies that contribute to the reduction of malaria.

Please read this document carefully and ask any questions you think may be relevant before deciding to participate in the study. We will explain the details of the study to you and leave a copy of this consent form for you to keep.

What will happen during the study?

This study will be carried out both in homes that decided to renovate their house by installing mosquito netting and also in those that have not decided to do so within the community of Llanchama. The measurement will be carried out before, after, six months and one year after the renewal.

You, as a Specialist in the human bait technique, will carry out the count of hematophagous vectors between 6 pm to 6 am, in the intra-domiciliary, peri-domiciliary and extra-domiciliary areas, then the information collected will be delivered to your supervisor from the Environmental Health Directorate of the Loreto Regional Health Management, so that they can calculate all the risk indicators of malaria transmission.

This information and others that will be collected in the community will help determine the findings related to the objectives of this study.

If you agree to assume this role during the study, you must sign this informed consent and present a certificate of your last training, which must be less than one year old.

Is there any benefit from participating in the study?

In addition to the professional experience that participating in this experimental study may provide you, as part of the partnership between GERESA Loreto, World Vision and KOICA, your participation and role will be mentioned in the study credits.

Will participating in the study have any risks?

As you are aware, applying the human baiting technique can put you at risk of contracting malaria, however, this risk is reduced as long as you apply the technique correctly and report any unforeseen events and incidents that you cannot report to your supervisor. have control and that puts your health at risk, to take any preventive measures in a timely manner.

Likewise, for your safety, you will be accompanied at all times by another person from the Environmental Health Directorate team or another person from the community who will support you while you execute the technique and will ensure that you are not in any danger if you have to do the count. in the intradomiciliary, peridomicile and extradomiciliary.

After having considered the information provided to me, I declare that:

I...

(Name and surname)

With DNI Nª: ....I declare that my decision is as follows:

☐Give☐I do not give

My consent to perform the human baiting technique, according to the conditions detailed in the information sheet.

SIGNATURE OF THE SPECIALIST IN HUMAN BAIT TECHNIQUE	SIGNATURE OF THE PERSON WHO ADMINISTERED THE CONSENT
NAME:	NAME:
DATE:	DATE:
