# Supplementary material for: Efficacy of ceiling-mounted mosquito nets for malaria vector control in a Peruvian Amazon riverine community: A stepped-wedge cluster randomized trial
Source: PLoS One. 2025 Oct 21;20(10):e0325089. doi: 10.1371/journal.pone.0325089 (PMC12539722; doi:10.1371/journal.pone.0325089)
Supplement: S2 File — This file provides detailed information on the study site and population characteristics in Llanchama, a rural Amazonian village in Loreto, Peru. It includes maps and drone images of the community layout, descriptions of local socioeconomic and environmental conditions, malaria transmission dynamics, and housing features relevant to vector exposure. The document also summarizes the implementation of CMMNs within the stepped-wedge cluster randomized trial, including structural modifications to typical Amazonian homes and baseline entomological context. (PDF) [file pone.0325089.s002.pdf]

S2 File. Study population, geographic context, and intervention setting

Characteristics of the Llanchama community

The study was conducted in **Llanchama** (Latitude: –3.8584, Longitude: –73.4119), a rural village located in the San Juan Bautista District of the Maynas Province, in the Loreto region of northeastern Peru. Llanchama is situated approximately 15 km south of **Iquitos** (Latitude: –3.8646, Longitude: –73.3250), the largest city in the Peruvian Amazon and one of the most remote urban centers in the world, accessible only by air or river. This region is highly endemic for **malaria**, with transmission of both *Plasmodium falciparum* and *Plasmodium vivax* occurring year-round and peaking during the **rainy season**, which extends from January through July.

Malaria Transmission and Control in Peru

In Peru, **malaria surveillance and treatment** fall under the responsibility of the **Ministry of Health (Ministerio de Salud del Perú, MoH)**. The national malaria strategy, updated most recently under the "**Malaria Zero**" initiative (2017–2030), emphasizes timely case detection, treatment, and vector control, with a focus on endemic regions such as Loreto.

Malaria cases are predominantly detected through **passive case surveillance**, in which symptomatic individuals self-present at community health posts or larger clinics. Diagnosis is performed by **microscopy**, with blood smears stained and read by trained MoH technicians. **Rapid diagnostic tests** are also available but are less commonly used in the Iquitos region due to continued reliance on microscopy-based confirmation.

Treatment is provided **free of charge** to all confirmed malaria cases, following **Peru's national malaria treatment guidelines**. These are consistent with WHO recommendations and include:

- For ***P. falciparum***: A combination of **artemether-lumefantrine (AL)** and a **single dose of primaquine** (0.75 mg/kg) to block transmission.
- For ***P. vivax***: A 3-day course of **chloroquine** (10 mg base/kg on day 1 and 2, 5 mg/kg on day 3), followed by a **14-day course of primaquine** (0.25 mg/kg/day) for radical cure, contingent on negative G6PD screening.
- Drug supplies are strictly managed by the MoH and distributed through regional health directorates. All malaria treatment is documented and reported through the **Sistema de Información en Salud (HIS-MINSA)**.

Description of the Zungarococha Community

Llanchama is one of four villages comprising the **Zungarococha community** (total population ~1907), along with **Zungarococha town**, **Puerto Almendra**, and **Ninarumi**. All four villages lie within a 2 km radius and are served by a single **Ministry of Health post located in Zungarococha town**, which serves as the main referral point for malaria diagnosis and treatment (Fig. S2A).

Figure S2A. Drone picture of the Llanchama community in the Loreto Region, Peru

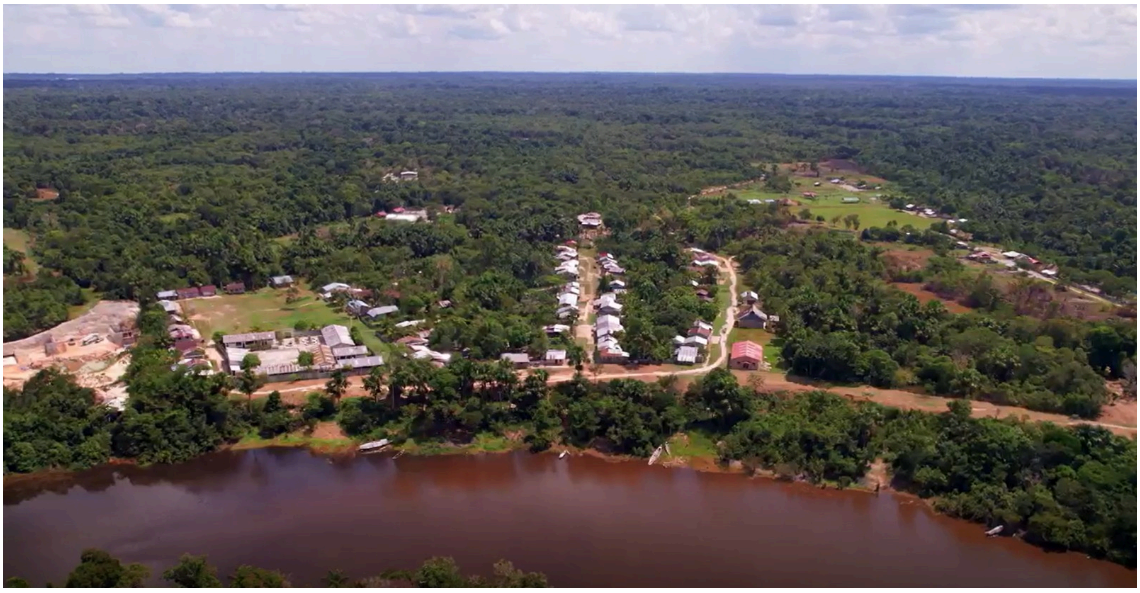

Source: World Vision Peru

The villages are accessible via a partially paved road extending from Iquitos; however, this road is often passable **only during the dry season** (August–December) and requires **four-wheel-drive vehicles**. During the rainy season, flooding and road damage often disrupt land transportation. A local **bus service** connects Llanchama with Zungarococha town, although its operation depends on road conditions.

Socioeconomic and Environmental Characteristics

Residents of Llanchama and surrounding villages engage primarily in **subsistence agriculture and fishing**. Men often work in agriculture, fishery, or manual labor (e.g., construction or farm maintenance) in commercial farms or the nearby **National Agrarian University of the Jungle (UNIA)**. Women are typically involved in household activities or informal local trade.

Most houses in Llanchama are built using **wooden frames with palm-thatched roofs**, typically lacking **window or door screens**, which allows mosquitoes easy indoor access. Homes are modest in size (average ~8 × 12 meters) and are built in **clusters along unpaved dirt roads**, with over 70% located less than six meters apart. Households usually face the street and open into **backyards rich in vegetation**, which are often within **100 meters of natural water bodies**. These riparian "backyard" areas are frequently used for cooking, washing, and socializing—activities that increase the risk of human-vector contact, particularly during peak mosquito biting hours at dusk and dawn.

The types of houses in Llanchama, Loreto, Peru, were classified into four types for the purpose of the study. These four types of houses included: (A) Type A, consisting of a thatched roof supported by wooden poles, with or without a raised wooden floor and no walls; (B) Type B, with a roof and partial wooden walls but without doors or windows, leaving the space semi-open; (C) Type C, fully enclosed with wooden walls and roof, featuring an open interior without divisions, doors, or windows; and (D) Type D, a more consolidated structure with a metal roof and ceiling, fully enclosed with walls, internal divisions, doors, and meshed windows for better protection (Fig. S2B)

The area is characterized by **high vector density**, particularly of *Anopheles darlingi*, the primary malaria vector in the region. Factors contributing to high vector exposure include the proximity of households to **marshlands, slow-flowing rivers, and ponds**, which serve as mosquito breeding sites.

The community lacks **electricity and running water**, so most activities are performed during daylight hours, and water is sourced from wells, rivers, or rainwater collection. **Health-seeking behavior** is generally driven by symptom severity and geographic proximity to the health post, which influences malaria case reporting.

Figure S2B. Household types A, B, C, and D at the Llanchama community

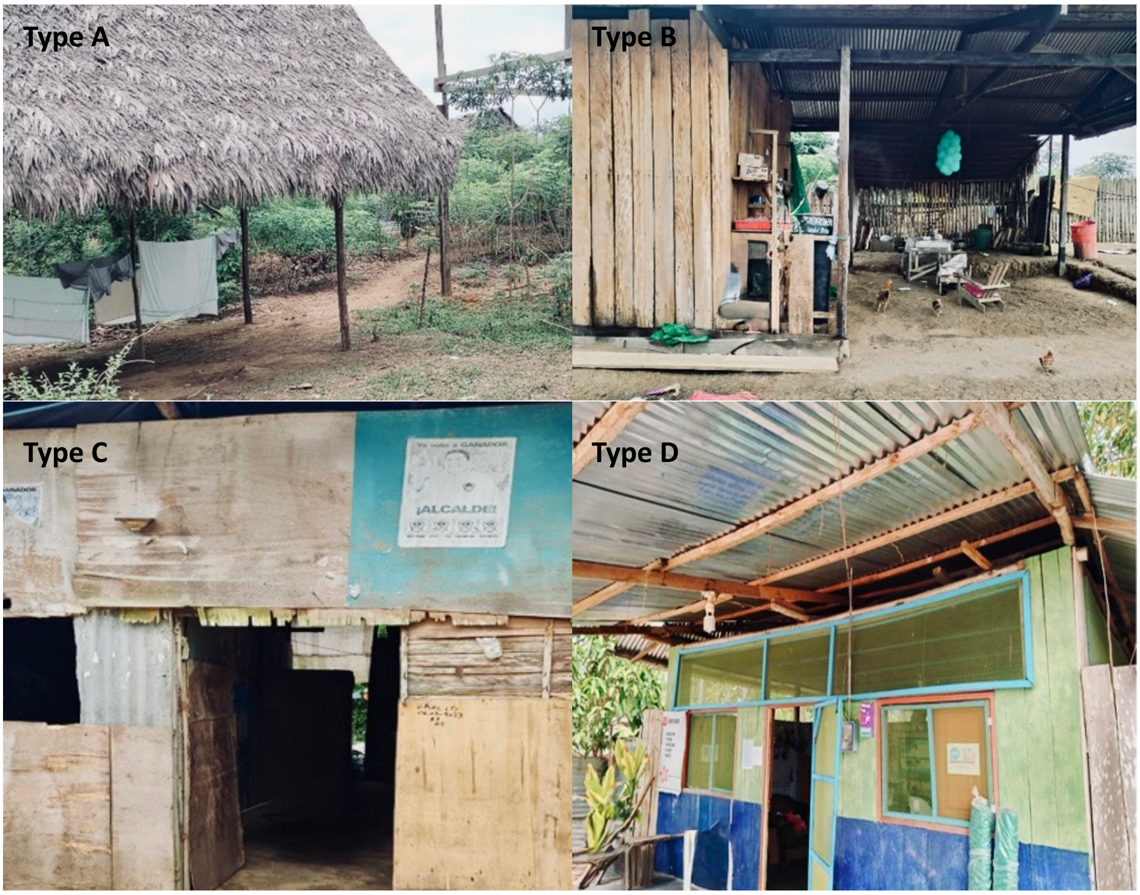

Source: Blgo. Freddy Gutierrez Rodriguez

Study Intervention

The intervention involved structurally modifying houses to install CMMNs and other open areas. This approach was designed to reduce mosquito entry, lower indoor Anopheles densities, and subsequently decrease malaria transmission risk. The remodeling process entailed retrofitting existing homes with durable, mosquito nets to create both a physical barrier against mosquitoes. Prior to remodeling, households were surveyed to document their structural characteristics, existing protective measures (e.g., bed nets, screens), and baseline entomological indicators (Fig. S2C).

The intervention was tailored to the typical housing structures found in the Peruvian Amazon, which often feature elevated wooden floors, open eaves, and palm-thatched or corrugated metal roofs. Homes with large cracks or exposed sections in wooden plank walls had these areas covered with mosquito netting to further reduce entry points. The netting material used was standard high-density polyethylene (HDPE) mosquito netting (0.28 monofilament with a fine mesh of ≥156 holes per square inch), designed to withstand humidity, ultraviolet exposure (UV protection of 600 kilo-light-years, equivalent to 4 years), and mechanical wear, thereby ensuring long-term protection.

The intervention was introduced in a phased manner across three clusters over nine months. Every three months, a new cluster of households received the materials and underwent remodeling, allowing for a controlled evaluation of the intervention's impact while ensuring that all households ultimately benefited.

Figure S2C. Ceiling-mounted mosquito nets at the Llanchama community

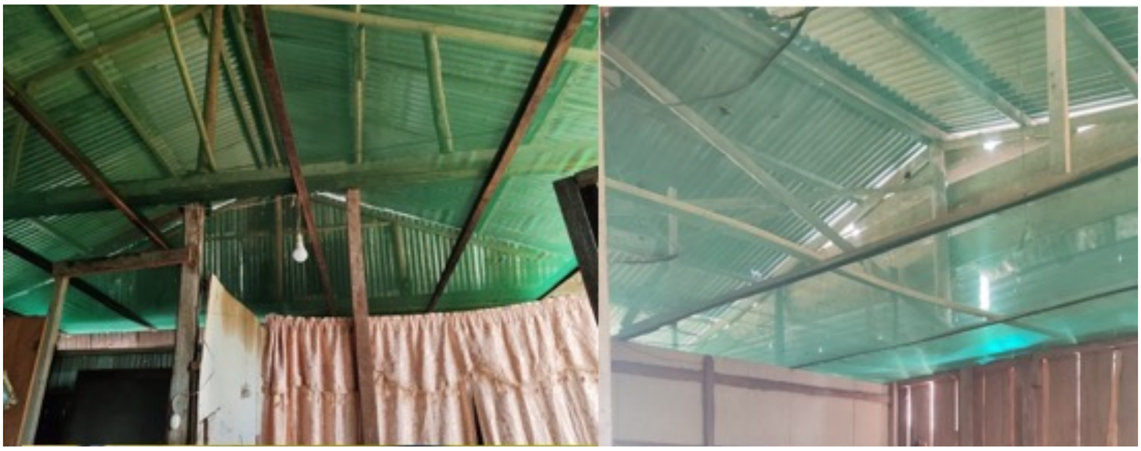

Source: World Vision Peru

Study outcomes

The study assessed the effectiveness of CMMNs in reducing mosquito density and human exposure, using standardized entomological methods. The primary outcomes were counts of Anopheles mosquitoes—captured indoors and outdoors via human landing catches (HLCs)—to quantify overall mosquito abundance and spatial distribution

within the community. Secondary outcomes focused on malaria transmission risk, measured as bites per person per night (BPN) and bites per person per hour (BPH), both indoors and outdoors.

Mosquito collections followed standard methods[1] and involved oral aspirators, collection jars, and flashlights. Briefly, teams collected mosquitoes indoors and outdoors (approximately 10 meters from the front door) over 12-hour periods (18:00 to 06:00) on two consecutive nights, rotating collectors every two hours to account for individual differences in attractiveness to mosquitoes.

Data from these 12-hour HLC sessions were used to calculate BPN and BPH. By comparing these entomological indicators before and after the intervention—and adjusting for the SW-RCT design—the study aimed to determine whether this structural modification effectively reduced the indoor presence of mosquitoes and, consequently, the potential for malaria transmission in a high-burden Amazonian community.

---

[1] Moreno M, Saavedra MP, Bickersmith SA, Lainhart W, Tong C, Alava F, et al. Implications for changes in Anopheles darlingi biting behaviour in three communities in the peri-Iquitos region of Amazonian Peru. Malar J. 2015;14:290. Epub 20150730. doi: 10.1186/s12936-015-0804-2. PubMed PMID: 26223450; PubMed Central PMCID: PMCPMC4518648.
