# Supplementary material for: Efficacy of ceiling-mounted mosquito nets for malaria vector control in a Peruvian Amazon riverine community: A stepped-wedge cluster randomized trial
Source: PLoS One. 2025 Oct 21;20(10):e0325089. doi: 10.1371/journal.pone.0325089 (PMC12539722; doi:10.1371/journal.pone.0325089)
Supplement: Attachment — Submitted filename: Reviewer comments_Sept 2025.pdf [file pone.0325089.s005.pdf]

Thank you for the chance to review the manuscript.

I have reviewed the manuscript and I see the authors have addressed all comments sufficiently and I recommend this manuscript to be published for it introduce one intervention that can be used to control malaria vectors and other mosquito-borne diseases

Thank you
