# Supplementary material for: Efficacy of ceiling-mounted mosquito nets for malaria vector control in a Peruvian Amazon riverine community: A stepped-wedge cluster randomized trial
Source: PLoS One. 2025 Oct 21;20(10):e0325089. doi: 10.1371/journal.pone.0325089 (PMC12539722; doi:10.1371/journal.pone.0325089)
Supplement: Attachment — Submitted filename: Review comments to authors.docx [file pone.0325089.s003.docx]

Thank you for the opportunity to review this manuscript. This is the good manuscript as it is portraying a new intervention that can be used to control mosquito-borne diseases especially indoor transmissions. Great work

Abstract

Statistical analysis (how the data were analyzed) is missing in the abstract section. It is important to appear in the abstract section

Line 40: I don’t think funding is part of the abstract. The authors should revise the journal submission guidelines if this adheres to these guidelines

Introduction section

Line 47-49 Authors have described the vector control tools such as ITNs and IRS. Could the authors describe the challenges that may be facilitating the persistence of malaria transmission despite the use of these traditional interventions?

The Introduction is well narrated

Materials and methods

The method section is well explained and it is sufficient. The study design, study procedures and data analysis section are well narrated. The authors have done a good job

Results

Line 265-280 should be moved to the methods section

Discussion

I have no comment in the discussion section, it is well written and it made the study very important.
